# Robust Stability of Scaled-Four-Channel Teleoperation with Internet Time-Varying Delays

**DOI:** 10.3390/s16050593

**Published:** 2016-04-26

**Authors:** Emma Delgado, Antonio Barreiro, Pablo Falcón, Miguel Díaz-Cacho

**Affiliations:** 1 School of Industrial Engineering, University of Vigo, 36310 Vigo, Spain; abarreiro@uvigo.es (A.B.); pfalcon@uvigo.es (P.F.); 2 School of Computer Engineering, University of Vigo, 32004 Orense, Spain; mcacho@uvigo.es

**Keywords:** teleoperation, robust stability, time-varying delays, transparency

## Abstract

We describe the application of a generic stability framework for a teleoperation system under time-varying delay conditions, as addressed in a previous work, to a scaled-four-channel (γ-4C) control scheme. Described is how varying delays are dealt with by means of dynamic encapsulation, giving rise to mu-test conditions for robust stability and offering an appealing frequency technique to deal with the stability robustness of the architecture. We discuss ideal transparency problems and we adapt classical solutions so that controllers are proper, without single or double differentiators, and thus avoid the negative effects of noise. The control scheme was fine-tuned and tested for complete stability to zero of the whole state, while seeking a practical solution to the trade-off between stability and transparency in the Internet-based teleoperation. These ideas were tested on an Internet-based application with two Omni devices at remote laboratory locations via simulations and real remote experiments that achieved robust stability, while performing well in terms of position synchronization and force transparency.

## 1. Introduction

A teleoperation system consists of master and slave mechanical systems where the master is directly manipulated by a human operator and the slave, operating in a remote environment, is designed to track the master closely. The main issues in the analysis and synthesis of these systems are stability and transparency. Transparency refers to the level of achievement of an ideal situation of the operator acting on the remote environment. In practice, there is a compromise between these two goals mainly due to the presence of time delays generated by the communication channel [1].

Given this compromise, many control schemes for teleoperation have been proposed, broadly classified in terms of two main frameworks, namely, intrinsically stable schemes (passivity-based), and delay-dependent stability schemes [2]. In early works on constant delay, stability was addressed by means of frequency, Laplace or passivity techniques applied to linear time-invariant (LTI) master-slave two-port systems [1,2,3,4,5].

In regard to transparency properties, the most successful control scheme in achieving full transparency under ideal conditions is the four-channel (4C) control scheme [1,2,4,6]. The specialized literature contains very detailed analyses of transparency and control architectures based on four, three and two channels, that, in general assume constant delays (see especially [4] and references therein).

However, in recent years a new line of research on Internet-based teleoperation [6,7,8,9,10,11] exposes the system control loop to the time-varying delay of a packet-switched network—the subject of a tutorial presented by Hokayem and Spong [12].

Control schemes designed within the passivity framework using scattering and two-port network theory concepts have been improved—by Chopra, Spong and Lozano [13]—by means of an adaptive coordination architecture that provides robust stability against constant delay in network and position tracking. Reset control techniques have also been used to enhance performance [14] and, more recently, passivity-based controllers have also been described that include scattering-based, damping injection and adaptive controllers [15].

Other research lines refer to time-delay compensation to improve teleoperation performance and stability, such as a passivity-based approach to constant delay [16] or methods based on the concept of network disturbance and the communication disturbance observer [9,17,18]. All these theoretical approaches assume a global delay given by τ = τ_m_ + τ_s_ (the sum of the delays between master and slave and vice versa) and a Laplace delay block e^−τs^ representing the global delay, which can commute with the other LTI blocks in the control loop. Therefore, although experimental results for simulated time-varying delay based on these techniques have been described [9,17,18], the stability condition for constant delay cannot apply. The loop must maintain all the time-varying delay channels of the control scheme because these cannot be combined in a single delay: time-varying delays do not commute with LTI systems.

Focusing on Internet-based teleoperation based on delay-dependent control schemes which can provide better transparency properties, the real problem is to establish delay-dependent conditions for systems with two internal control loops (master and slave) ”connected” by (time-varying) delayed signals. This problem can be solved—to name just two possible approaches—by applying delay-dependent stability tools developed for time-domain techniques that are based on Lyapunov-Krasovskii functionals, with a formulation of stability obtained via easily computable LMI conditions (see [6,10,19,20] and references therein), or by using approaches based on small-gain type theorems in the input-to-state stability framework [21], which can be used even when passivity is lost.

More specifically, referring to previous research by us, focusing on the stability of the delay-dependent teleoperation control schemes, we defined a generic approach to modelling any teleoperation setup as a negative single-feedback loop containing an LTI block and an uncertain time-varying delay [11]. The main contribution of this approach was the possibility of deriving frequency-domain conditions for robust stability in the presence of time-varying delays and parametric uncertainties. As a case study, the two-channel position-error (PE) control scheme was modelled, implemented and experimentally tested for the Internet-based haptic teleoperation of a laboratory 3D crane in normal operation conditions, that is, without contact with the environment.

Using this generic approach, we presented preliminary results [22] on the 4C control scheme for teleoperating simulated manipulators, as more flexible than 3C [23] or 2C [24] versions. Further work [25] addressed the robust stability of simulated teleoperated systems, with the 4C architecture affected by time-varying communication delays, by using disturbance observers that are relevant when only position (but not force) measurements are available.

The main differences between our approach [6,22,25] and other delay-dependent proposals are that we look for: (a) complete stability to zero of the whole state; and (b) robustness against continuous-time time-varying delay. The first goal implies that, in the absence of external inputs, the whole state should converge to zero. In particular, the nominal, non-delayed closed-loop system should have well-posed dynamics dx/dt = Ax, with stable closed-loop eigenvalues, Real(Eig(A)) < 0. This excludes schemes where master and slave positions mutually converge x_m_ → x_s_ but do not tend to zero. It also excludes cases where the master or the slave ports should be closed by dissipative port terminations to ensure stability. The first goal is important in applications where master or slave devices could be released and we want the resulting free motion to tend to zero (a fixed safe position in the workspace).

It is well-known [4] that 4C schemes with perfect transparency do not have well-posedness and complete stability to zero of the state vector. This is the reason why, starting out with the 4C architecture [6,22,25], we introduced a scaling parameter γ to achieve complete stability to zero of the whole state.

The second goal is to ensure robust stability in Internet-based teleoperation, that is, under time-varying delay. We address the problem of uncertain delays from a continuous-time perspective (not in discrete-time [26,27]) and for time-varying delays (more realistic than uncertain but constant delays [28]). The induced problem is more difficult but, since useful delay encapsulations have been described in the literature [29], we show how to adapt these delay bounds to achieve our second goal of robustness against continuous-time time-varying delay.

The main differences between our previous work and the present work—in other words, the novelty of the present paper—is that here we explicitly discuss ideal transparency issues and rearrange previous solutions [22,25] so that now the resulting controllers are proper, that is, they do not contain single or double differentiators and so avoid problems of noise. We also validated the ideas with simulations and real-life experiments on an Internet teleoperation setup that coordinated two Omni haptic devices between remote laboratory setups in Spain (University of Vigo, Vigo, Spain) and the UK (University of Manchester, Manchester, UK).

The paper is organized as follows: in Section 2 the model for robust stability in 4C-based teleoperation is introduced and in Section 3 a robust stability condition for this control scheme is defined. Section 4 describes the haptic teleoperation case study. Section 5 presents the analysis and simulation results and Section 6 presents the experimental results. Finally, Section 7 concludes the paper with a discussion.

## 2. Model for Robust Stability in 4C Based Teleoperation

**Figure 1:**
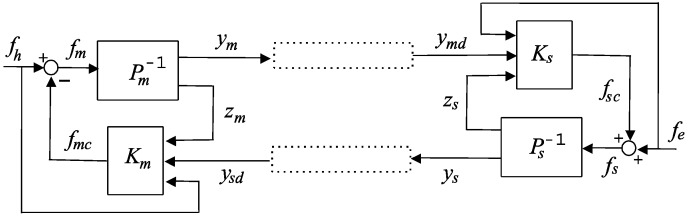
Generic teleoperation system.

Consider the generic teleoperation setup depicted in Figure 1, consisting of an exchange of signals through a communication channel between the master side (left) and the slave side (right). The master side is formed of the plant 1/*P_m_* and the controller *K_m_*. The plant is modelled as a mechanical system with coordinates (positions) given by the *n* × 1 vectors *x_m_*, *x_s_*. In the Laplace domain, if $x_{m}(0)={\dot{x}}_{m}(0)=0,$ we have:

(1) $$P_{m}(s)X_{m}(s):=F_{m}(s),P_{s}(s)X_{s}(s):=F_{s}(s)$$

Note that we use lowercase and uppercase letters for time-domain and Laplace domain signals, respectively.

The input of the master plant is the master force $f_{m}=f_{h}-f_{mc},$ where *f_h_* is the force applied by the human operator and *f_mc_* is the force from the controller. The plant has two outputs, namely, the *n_y_* signals that send information to the slave (*y_m_*) and the *n_z_* signals used by the local controller (*z_m_*). 

On the slave side, *f_e_* is the force from the environment. The controller force *f_sc_* enters additively in $f_{s}=f_{e}+f_{sc}.$

Master and slave exchange signals through the communication channel. Taking into account our theoretical [10], and experimental [11] discussions on delay bounds using the Internet User Datagram Protocol (UDP), the delay τ(t) can be assumed in a non-restrictive way from teleoperation point of view, to be an unknown time-varying function for which the upper bounds on the magnitude and the variation satisfy ∀t≥0:

(2) $$0\leq \tau (t)=h+\eta (t)\leq h_{\max},\begin{array}{cc} & \left|\eta (t)\right| \end{array}\leq \kappa \leq h$$

(3) $$\left|\dot{\tau }(t)\right|\leq d<1$$

The time-varying delay can be described by the operator *𝒟* which is a (nonlinear) system used (in the time domain) in any of the equivalent notations: $y_{jd}=D_{j}(y_{j})=D_{j}\circ y_{j}=D_{j}\;y_{j},\;\;(j=m,s)$. This means that:

(4) $$y_{md_{i}}(t)=D_{m}y_{m_{i}}(t):=y_{m}{}_{i}(t-\tau_{m}(t)),\begin{array}{cc} & y_{sd_{i}}(t)=D_{s}y_{s_{i}}(t):=y_{s}{}_{i}(t-\tau_{s}(t)) \end{array}$$

where *𝒟 _m_*, *𝒟 _s_* are the corresponding master and slave time-delay operators. To simplify matters we also extend the operator *𝒟* to the Laplace domain in the equivalent forms $Y_{jd}=D_{j}(Y_{j})=D_{j}\circ Y_{j}=D_{j}\;Y_{j}$, which simply means that: $Y_{jd}(s)=D_{j}\;Y_{j}(s)\Leftrightarrow Y_{jd}(s)=L\left(y_{jd}(t)\right)$ and $y_{jd}(t)=D_{j}\;y_{j}(t)$.

**Figure 2:**
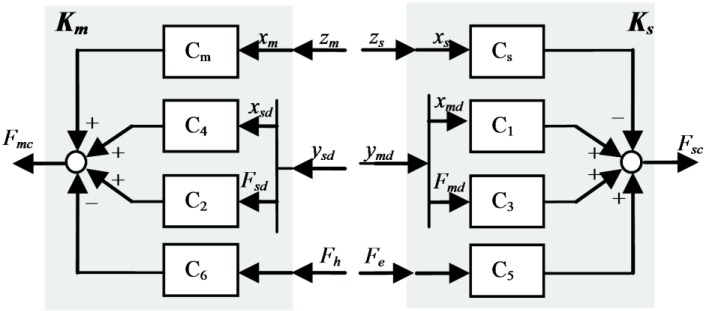
Local controllers in the 4C control scheme.

We now consider a 4C control scheme in the most general and representative case of delay-dependent schemes. Here, the master and slave exchange, through the communication channel, velocities or positions and forces in both directions. The local controllers are defined in terms of the applied external force, their own velocity/position (master/slave) and the delayed velocity/position and force (slave/master) from the other side. When the system exchanges positions and forces (see Figure 2), the controllers *K_m_*, *K_s_* can be defined as:

(5) $$f_{mc}=C_{m}x_{m}+C_{2}f_{sd}+C_{4}x_{sd}-C_{6}f_{h}$$

(6) $$f_{sc}=-C_{s}x_{s}+C_{3}f_{md}+C_{1}x_{md}+C_{5}f_{e}$$

**Figure 3:**
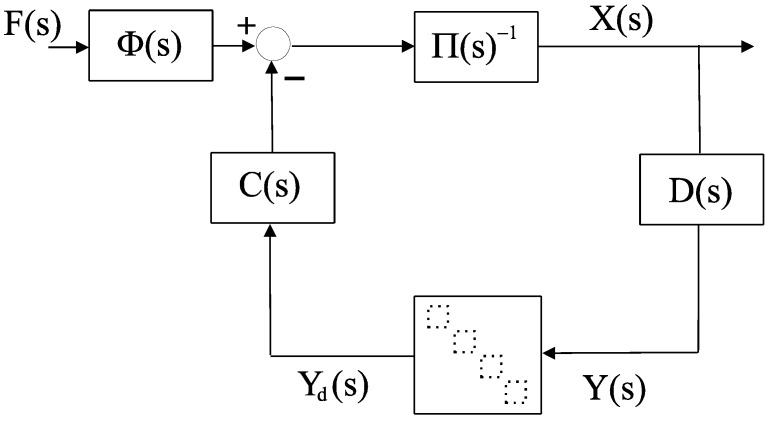
Teleoperation loop.

Following the procedure described in a previous work [11], we remodeled this whole system to obtain a single feedback loop containing an LTI block and an uncertain time-varying delay block with the same dimension as the number of the signals exchanged between the master and slave. For this purpose, we state the following Proposition 1 to obtain the model for 4C-based teleoperation:

**Proposition 1.**  A teleoperation setup as described in Figure 1 and Figure 2, given by Equations (1)–(5), can be formulated as a compact model for robust stability analysis using a generic approach (as developed in [11]) in the form given by equations Equation (7), Equation (8) and the block diagram in Figure 3:

(7) Π(s)X(s)=−C(s)DD(s)X(s)+Φ(s)F(s)

with:

(8) $$\begin{array}{l} \Pi =\left(\begin{array}{cc} P_{m}+C_{m} & 0\\ 0 & P_{s}+C_{s} \end{array}\right),\;X=\left(\begin{array}{c} X_{m}\\ X_{s} \end{array}\right),\;C=\;\left(\begin{array}{cc} 0_{1\times 2} & \begin{array}{cc} C_{4} & C_{2} \end{array}\\ \begin{array}{cc} {-\text{C}}_{1} & {-\text{C}}_{3} \end{array} & 0_{1\times 2} \end{array}\right),D=\left(\begin{array}{cc} I_{2}\cdot D_{m} & 0_{2\times 2}\\ 0_{2\times 2} & I_{2}\cdot D_{s} \end{array}\right),\;\\ \;D=\left(\begin{array}{cc} {\left(\begin{array}{cc} 1 & P_{m} \end{array}\right)}^{T} & 0_{2\times 1}\\ 0_{2\times 1} & {\left(\begin{array}{cc} 1 & P_{s} \end{array}\right)}^{T} \end{array}\right),\;\Phi =\left(\begin{array}{cc} 1+C_{6} & 0\\ 0 & 1+C_{5} \end{array}\right),\;F=\left(\begin{array}{c} F_{h}\\ F_{e} \end{array}\right) \end{array}$$

**Proof:**  The proof of this proposition is developed in Appendix A. ☐

**Remark 1.**  The model described by Equation (7) is directly represented by Figure 3, where four time-varying delays channels inherited from Figure 1 are maintained, bearing in mind that they cannot be combined in a single delay because time-varying delays do not commute with LTI systems.

### Ideal Transparency Conditions

Note that, from the teleoperation loop in Figure 3, we can also obtain the zero-delay (*D* = *I* ⇒ *Y_d_* *=* *Y*) closed loop dynamics for studying ideal behavior and transparency properties, considering:

(9) $$X(s)={\left(\Pi (s)+C(s)D(s)\right)}^{-1}\Phi (s)F(s)=:\Lambda (s)F(s)$$

That is:

(10) $$\left(\begin{array}{c} x_{m}\\ x_{s} \end{array}\right)=\Lambda \cdot \left(\begin{array}{c} f_{h}\\ f_{e} \end{array}\right)\Rightarrow \left\{ \begin{array}{l} x_{m}=\Delta_{\Lambda }{}^{-1}\cdot (\Lambda_{11}f_{h}+\Lambda_{12}f_{e})\\ x_{s}=\Delta_{\Lambda }{}^{-1}\cdot (\Lambda_{21}f_{h}+\Lambda_{22}f_{e}) \end{array}\right.$$

with the following values:

(11) $$\Lambda_{11}=\left(P_{s}+C_{s}\right)\cdot \left(1+C_{6}\right),\;\Lambda_{12}=-\left(C_{4}+C_{2}P_{s}\right)\cdot \left(1+C_{5}\right)\;\Lambda_{21}=\left(C_{1}+C_{3}P_{m}\right)\cdot \left(1+C_{6}\right),\;\Lambda_{22}=\left(P_{m}+C_{m}\right)\cdot \left(1+C_{5}\right)\;\Delta_{\Lambda }=\left(P_{m}+C_{m}\right)\cdot \left(P_{s}+C_{s}\right)+\left(C_{1}+C_{3}P_{m}\right)\left(C_{4}+C_{2}P_{s}\right)$$

The previous admittance matrix definition can be associated with the impedance *𝒵* or hybrid *ℋ* models:

(12) $$\left(\begin{array}{c} f_{h}\\ f_{e} \end{array}\right)=Z\cdot \left(\begin{array}{c} x_{m}\\ x_{s} \end{array}\right)\Rightarrow \left\{ \begin{array}{l} f_{h}=\Delta_{Z}{}^{-1}\cdot \left(\Lambda_{22}x_{m}-\Lambda_{12}x_{s}\right)\\ f_{e}=\Delta_{Z}{}^{-1}\cdot \left(-\Lambda_{21}x_{m}+\Lambda_{11}x_{s}\right) \end{array}\right.,\;\Delta_{Z}=\left(1+C_{5}\right)\cdot \left(1+C_{6}\right)$$

(13) $$\left(\begin{array}{c} f_{h}\\ x_{s} \end{array}\right)=H\cdot \left(\begin{array}{c} x_{m}\\ f_{e} \end{array}\right)\Rightarrow \left\{ \begin{array}{l} f_{h}=h_{11}x_{m}+h_{12}f_{e}\\ x_{s}=h_{21}x_{m}+h_{22}f_{e} \end{array}\right.$$

The ideal transparency properties are usually defined on the ℋ matrix, regarding which:

(14) $$h_{11}=h_{22}=0\;transparency,\;h_{12}\cdot \;h_{21}=-1\;condition\;set$$

Comparing Equations (13) and (14) with Equation (9), we obtain:

(15) $$h_{11}\to 0\;\Rightarrow \;\Delta_{\Lambda }\to 0\;\;,\;\;h_{12}=-\frac{\Lambda_{12}}{\Lambda_{11}}=-1\Rightarrow \;\Lambda_{12}=\Lambda_{11}\;\;\;h_{21}=\frac{\Lambda_{21}}{\Lambda_{11}}=1\Rightarrow \Lambda_{21}=\Lambda_{11}\;,\;h_{22}=0\Rightarrow \Lambda_{11}\Lambda_{22}-\Lambda_{12}\Lambda_{21}=0\Rightarrow \Lambda_{22}=\Lambda_{11}$$

Therefore, these properties can also be analyzed through Λ and *𝒵* matrix in the following form:

(16) $$Transparency\;and\;Condition\;set\equiv \;Z_{ideal}=\frac{1}{\Delta_{Z}}\left(\begin{array}{cc} \Lambda_{11} & -\Lambda_{11}\\ -\Lambda_{11} & \Lambda_{11} \end{array}\right),\;\Lambda_{ideal}=\frac{1}{\Delta_{\Lambda }}\left(\begin{array}{cc} \Lambda_{11} & \Lambda_{11}\\ \Lambda_{11} & \Lambda_{11} \end{array}\right)\;with\;\Delta_{\Lambda }\to 0\;$$

## 3. Robust Stability for 4C Based Teleoperation

Below we apply our previously developed approach to stability under time-varying delay in teleoperation [11,22] to the 4C control scheme. The time-varying delay is an uncertainty that may give rise to instability. To ensure robust stability, of necessity we have to impose some limits on maximum delay and maximum delay variation. Our treatment of time-varying delay is based on an encapsulation described in the literature [29] and adapted by us to a 4C teleoperation setup. This delay encapsulation [29]—based on the concept of the integral quadratic constraint (IQC), which is a powerful framework for robust stability—is combined with input-output stability criteria and μ-analysis and synthesis techniques in order to achieve a final robust stability condition (Theorem 1).

**Figure 4:**
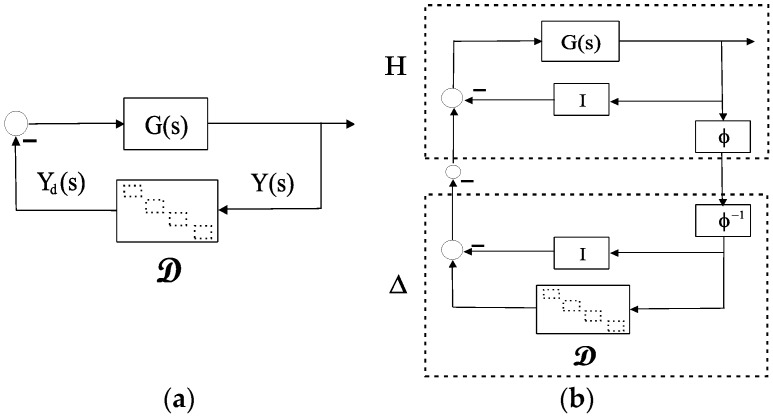
(**a**) LTI system with time-varying delay in the feedback loop; (**b**) Loop transformation.

**Proposition 2.**  A teleoperation setup, as stated in Proposition 1 and depicted in Figure 3, can be modeled as a negative single-feedback loop containing an LTI block *G(s)* and an uncertain time-varying delay block *𝒟* , shown in Figure 4a, with:

(17) $$G(s)=D(s)\Pi^{-1}(s)C(s)$$

(18) $$G=\left(\begin{array}{cc} 0_{2\times 2} & \begin{array}{cc} {\left(P_{m}+C_{m}\right)}^{-1}C_{4} & {\left(P_{m}+C_{m}\right)}^{-1}C_{2}\\ P_{m}{\left(P_{m}+C_{m}\right)}^{-1}C_{4} & P_{m}{\left(P_{m}+C_{m}\right)}^{-1}C_{2} \end{array}\\ \begin{array}{cc} -{\left(P_{s}+C_{s}\right)}^{-1}C_{1} & -{\left(P_{s}+C_{s}\right)}^{-1}C_{3}\\ -P_{s}{\left(P_{s}+C_{s}\right)}^{-1}C_{1} & -P_{s}{\left(P_{s}+C_{s}\right)}^{-1}C_{3} \end{array} & 0_{2\times 2} \end{array}\right)$$

**Theorem 1.**  *Consider a 4C-based teleoperation system, modeled by Equations (7) and (8), directly represented by Figure 3 and transformed into Figure 4a by Proposition 2. Given G(s) as defined in Equation (15), let φ(s) [29]:*

(19) $$\varphi (s)=k\cdot \frac{h_{\max}^{2}s^{2}+c\cdot h_{\max}s}{h_{\max}^{2}s^{2}+ah_{\max}s+k\cdot c}$$

with $k=1+1/\sqrt{1-d},a=\sqrt{2kc}$ where c is any positive real number and delay bounds *h_max_* and where *d* is as defined in Equations (2) and (3). If necessary, [29] provides extensions of the delay encapsulations by another multipliers φ in Equation (19) that deal with great variability. A sufficient condition for stability of the delayed system as described in Figure 4a, with a complex diagonal structured uncertainty, is given by:

(20) $$\Gamma =\underset{\omega \in \left[0,\infty \right]}{\max}\bar{\mu }\left[H(j\omega )\right]=\underset{\omega \in \left[0,\infty \right]}{\max}\rho \left[H(j\omega )\right]<1$$

where:

(21) $$H(s)=:\varphi (s)\cdot {\left(I+G(s)\right)}^{-1}G(s)$$

and where $\bar{\mu }\left(\cdot \right)$ is the structured singular value (SSV) with respect to a repeated complex scalar uncertainty, which is, in turn, equal to ρ(⋅), the spectral radius of a matrix (the maximum of the norms of the eigenvalues).

**Proof.**  This result is obtained in two ways. First, in applying the loop transformation theorem (the loop depicted in Figure 4a is stable if the loop depicted in Figure 4b is stable) and the small gain theorem to Figure 4b, whereby${\left\|H(s)\right\|}_{L_{2}}\cdot {\left\|\Delta \right\|}_{L_{2}}<1$, since ${\left\|\Delta \right\|}_{L_{2}}\leq 1$ by [29], a sufficient condition is ${\left\|H(s)\right\|}_{L_{2}}<1$. Second, using the μ-techniques for robust stability [30] we can interpret Figure 4b as a nominal system *H(s)* connected to a dynamic or complex uncertainty Δ that is actually diagonal (more details are given in [11]). ☐

**Remark 2.**  As a prerequisite for robust stability, we must first satisfy nominal stability. Hence, H(s) must be Hurwitz-stable, that is, all the λ_i_ eigenvalues must verify $real\left(\lambda_{i}\right)<0\begin{array}{cc} & \forall i,i=1\ldots n \end{array}$.

This frequency approach based on IQCs combined with μ-analysis and synthesis techniques is a powerful framework for robust stability. Therefore, using the stability condition of Equation (20) based on the system in Equation (21), for any delay satisfying Equations (2) and (3) and for some designed closed-loop (non-delayed) dynamic in G, we can ensure robust stability under time-varying delays.

## 4. Haptic Teleoperation Case Study

The previous 4C architectural framework was applied to remote teleoperation between master and slave haptic devices located in laboratories in the University of Vigo and the University of Manchester. 

Below we describe the details of the setup and of master and slave device identification. We then describe a scaled control scheme γ-4C in which the stability factor γ lets us obtain a practical and internally stable solution to Internet-based teleoperation, where all the controllers are proper (without differentiators), as is usual in classical solutions. Further sections will describe simulations and experimental results.

The master and slave sides consist of two Phantom Omni haptic devices manufactured by SensAble Technologies Inc. (Wilmington, MA, USA). In terms of degree of freedom (DoF), these devices have 6-DoF position sensing and 3-DoF force actuation. To focus on the robust stability issue, we limited movements to 1-DoF—rather than multi-DoF—using the first rotational coordinate (the base rotation) as the free DoF. In this way, the two Omnis rotated around their vertical axes, while the shoulder, elbow and stylus were blocked.

Thus, the master and slave positions were (*x_m_*, *x_s_*), the sensed Omni base angles in radians (rad). The actuations are (*f_m_*, *f_s_*), which are the torque inputs to the motors for vertical axis rotation in machine units (we did not have access to physical units).

We concentrated on 1-DoF movements around a fixed position (the “zero” of the Omnis). Although the Omnis are robotic nonlinear systems, given the linearization principle, it was expected that the local dynamics from forces to positions could be captured as is usual by models in the form *G_i_*(s) = 1/(s(*M*s + *B*)) [31]. Note that here, for simplicity sake, we use the terms “forces” and “positions” to denote torques and angles. Note also that, since the forces are in machine units, the inertia-mass *M* and friction *B* coefficients were given in machine or virtual units.

Before identifying the models *G_i_*(s), our experiments showed that there was a deadzone or static friction at the force inputs. This meant that the effective force *f_e_*_f_ was smaller than the applied or written force *f_i_*. This effect can be modelled by a deadzone function *f_ef_* = DZ(*f_i_*; *D_pos_*, *D_neg_*) in the form:

(22) $$f_{ef}=\underset{}{\max\left\{\begin{array}{cc} 0, & f_{i}-D_{pos} \end{array}\right\}}\;\begin{array}{cc} if & f_{i}>0, \end{array}f_{ef}=\underset{}{\min\left\{\begin{array}{cc} 0, & f_{i}+D_{neg} \end{array}\right\}}\;\begin{array}{cc} if & f_{i}<0 \end{array}$$

Although this static friction slightly damaged linearity, producing small steady-state errors, this was easily mitigated by pre-compensation at input by an inverse function in the form *f_i_* = DZ(*f*; −*D_pos_*, −*D_neg_*) with negative values −*D_pos_*, −*D_neg_*. If the deadzone (asymmetric) widths *D_pos_*, *D_neg_* were estimated well, the composition of these two functions would give rise to the identity, *f_ef_* = *f*, thus linearizing the system.

After linearizing the devices by deadzone compensation, the linear models *G_i_*(s) = 1/(s(*M*s + *B*)), were obtained, by a standard procedure [24] applied in closed -loop (proportional controller) square and triangular references, recording force inputs and position outputs and performing standard least-squares identifications of *M*, *B*. The results (in machine units, m.u.) were:

(23) *M* = 0.00118 (m.u.) *B* = 0.0199 (m.u.)

Therefore, for the teleoperation setup described in Figure 1 formed of two identical OMNI Phantoms as the master and slave systems, for each DoF, the linearized dynamic models were:

(24) $$P_{m}=Ms^{2}+Bs=P_{s}=P$$

Seeking a practical solution to Internet-based teleoperation, we fine-tuned a scaled 4C control scheme γ-4C. We also imposed internal stability, that is, the closed-loop system had to be Hurwitz-stable. The operator and environment were modelled as external forces.

These requirements were met in the scheme depicted in Figure 1 and Figure 2 and given by Equations (1)–(5), with master and slave systems as in Equation (24) and with the controllers in Equation (5) defined in the following form:

(25) $$For\;C_{m}=C_{s}=K>0\;\mathrm{and}\;\gamma >1:\;C_{4}(s)=-\frac{C_{s}}{\gamma },\begin{array}{cc} & \;C_{1}(s)=C_{m}, \end{array}\;\begin{array}{cccc} C_{2}=-\frac{1}{\gamma }, & C_{3}=1, & \;C_{5}=C_{6}=K\prime & \end{array}$$

The Λ and *𝒵* matrix in this γ-4C scheme with controllers given by Equation (25) take the values:

(26) $$\begin{array}{l} \Lambda (s)=\frac{\left(1+K\prime \right)}{\Delta_{\Lambda }}\left(\begin{array}{cc} \left(P+K\right) & \frac{1}{\gamma }\cdot \left(P+K\right)\\ \left(P+K\right) & \;\left(P+K\right) \end{array}\right),\begin{array}{cc} & \Delta_{\Lambda }={\left(P+K\right)}^{2}\cdot \left(1-\frac{1}{\gamma }\right) \end{array}\\ Z=\frac{1}{{\left(1+K\prime \right)}^{2}}\left(\begin{array}{cc} \left(P+K\right) & -\frac{1}{\gamma }\left(P+K\right)\\ -\left(P+K\right) & \left(P+K\right) \end{array}\right) \end{array}$$

Note that the control parameters in Equation (5) with the values selected in Equation (25) were not eight independent controllers. Due to the transparency restriction requirements, there were ultimately only a minimum of *two* DoFs in the design: the gain *K* and the coefficient γ (*K*’ can be zero). The gain *K* was the only parameter that directly influenced the closed-loop poles (Δ_Λ_ = 0) in the non-delayed case, as seen in Equation (26). The value *k* > 0 was necessary to ensure non-delayed system stability. We could thus tune *k* > 0 from pole-placement principles, bearing in mind that the feedback loop would be affected by a (time-varying) delay. As for the coefficient γ, this ideally should be set to γ = 1 to ensure perfect transparency, but to avoid singularity in the feedback loop impedance (admittance) matrix, it was set to γ > 1. For greater transparency, γ should be as close as 1 as possible, but has to be greater than 1 to ensure robust stability and loop well-posedness.

**Remark 3.**  If γ = 1 + ε, with ε → 0, the teleoperation system with the controllers defined by Equation (25) verify the transparency-optimized conditions for zero delay Equation (16). But in the ideal case ε = 0, the impedance matrix Equation (12) loses rank and thus the admittance model Equation (10) is thus not well-defined (ill-posed).

**Remark 4.**  To avoid the previous issue, the γ factor in Equation (25) can be selected to verify well-posedness and robust stability conditions under Internet time-varying delay, while maintaining the transparency properties of the system as much as possible. This control scheme therefore gives internal stability and a practical solution to the trade-off between performance and stability under time-varying delay.

**Remark 5.**  The proposed control scheme in Figure 1 and Figure 2 as given by Equation (5) allows proper controllers in Equation (25) to be selected based on ideally optimized transparency, that is, they do not contain single or double differentiators like classical 4C solutions and so avoid negative effects with noise. See the explanation in Appendix B.

**Remark 6.**  The controllers defined in Equation (25) also provide robust stability and performance against parametric uncertainty. The closed-loop poles are now given by (M_m_s^2^ + B_m_s + K) (M_s_s^2^ + B_s_s + K) = 0 and, consequently, for zero delay, the system will be stable iff K, M_m,s_ = M ± Δ_M_, B_m,s_ = B ± Δ_B_ >0.

These remarks are further analyzed in the following sections.

## 5. Analysis and Simulation Results

We first describe a simulation case for use with Simulink and Matlab, considering linear dynamic models for the master and slave, the rounded identified values given by *M* = 0.001, *B* = 0.02 and the controllers as stated in Equation (25), with *K* = 0.1 and *K’* = 0.

To justify choosing this control scheme based on performance criteria, using this simulation case and applying zero delay, we compared our scheme with some frequently used two- and three-channel (2C and 3C) control schemes. These schemes can be obtained by canceling certain controller parameters in Equation (25), as shown in Table 1.

Note that the 4C scheme results were obtained for γ = 1.07 ≈ 1 to avoid singularities. The selected value in *K* provided an adequate time response by fixing equal and real closed-loop poles, that is, the linear model of the system had critical damping and so did not overshoot.

Performance can be studied by simulations that apply a simulated sine reference force (*f_h_*)—like the force imposed by a human operator—to move the master. The slave will follow the master in free motion in the interval *t* = (0, 10) and (18, 30) s and with an environment step force *f_e_* simulated from *t* = 10 s and *t* = 18 s Note that the operator and environment are not assumed to be in contact or to be passive. 

**Figure 5:**
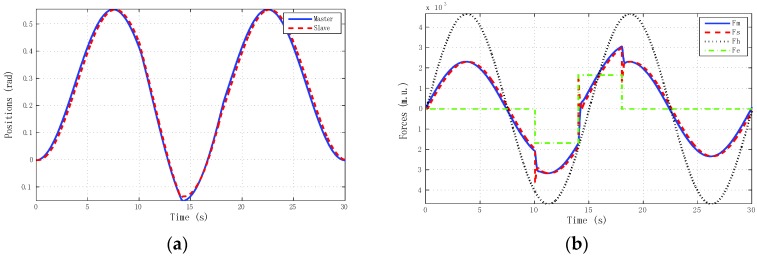
Simulated sine reference force: PE control scheme with hmax = 0, d = 0. (**a**) Positions; (**b**) Forces.

**Figure 6:**
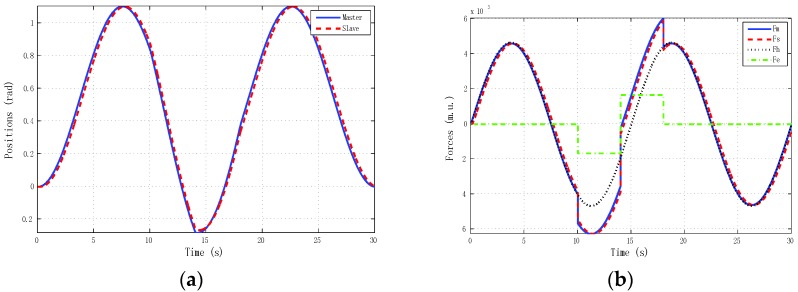
Simulated sine reference force: 3C-i control scheme with *h_max_* = 0, *d* = 0. (**a**) Positions; (**b**) Forces.

**Figure 7:**
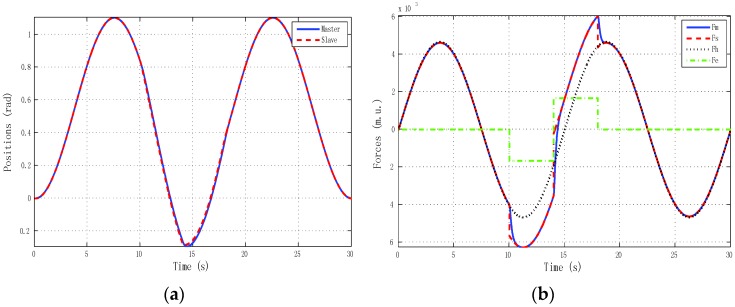
Simulated sine reference force: 3C-ii control scheme with *h_max_* = 0, *d* = 0. (**a**) Positions; (**b**) Forces.

**Figure 8:**
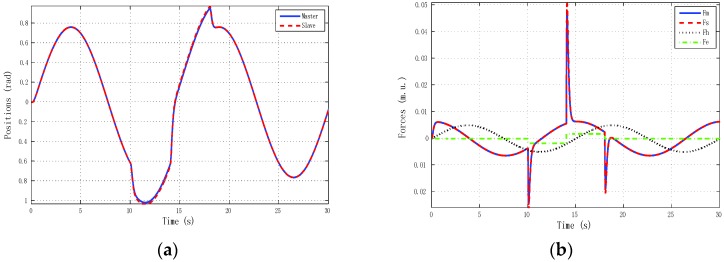
Simulated sine reference force: γ-4C control scheme with *h_max_* = 0, *d* = 0. (**a**) Positions; (**b**) Forces.

**Figure 9:**
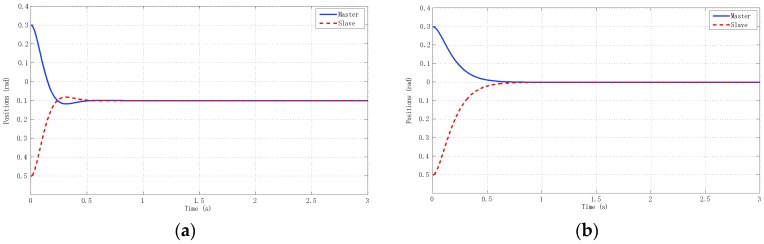
Simulated f_h_ = f_e_ = 0 with *h_max_* = 0, *d* = 0. Positions. (**a**) PE; (**b**) γ-4C.

The best results for tracking properties were obtained for the schemes which approximated the ideal transparency conditions (γ-4C) or, at least, the schemes (PE, 3Ci, 3Cii) in which conditions were achieved in the steady state (*s* → 0). The simulation results, for brevity, are shown only for these schemes (Figure 5, Figure 6, Figure 7 and Figure 8). Only the γ-4C scheme provided internal stability to the system. Because the other schemes have a closed-loop pole at the origin, without external forces, some states of the system tended to the same value (in this case *x_m_* = *x_s_*) but not to zero, for example, PE in Figure 9a. However, the γ-4C scheme in Figure 9b, without external forces, drove the master and slave to their zero states.

**Figure 10:**
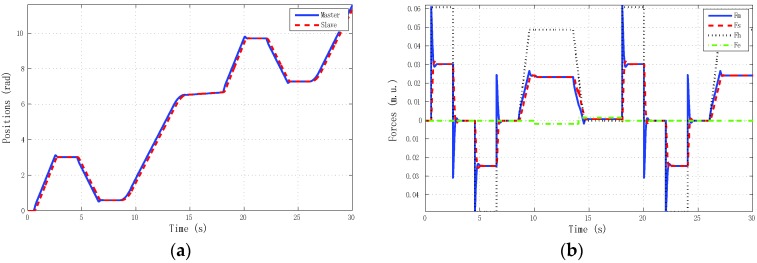
Simulated ramp-step reference force: PE control scheme with *h_max_* = 0, *d* = 0. (**a**) Positions; (**b**) Forces.

**Figure 11:**
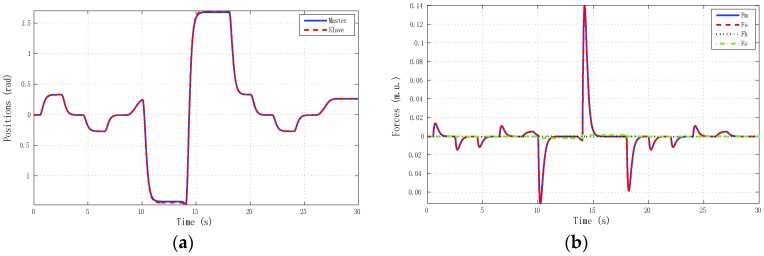
Simulated ramp-step reference force: γ-4C control scheme with *h_max_* = 0, *d* = 0. (**a**) Positions; (**b**) Forces.

Furthermore, it may happen that, for some input forces as used in remote teleoperation, the master and slave positions are not bounded for systems without internal stability, although position tracking is accomplished—for instance, one of the schemes in Figure 10 and the solution given for the 4C scheme in Figure 11.

**Figure 12:**
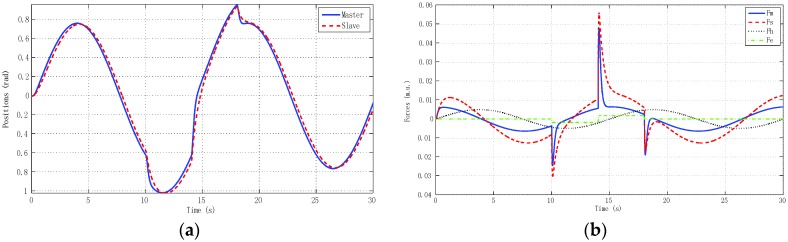
Simulated sine reference force: γ-4C control scheme, Ps ≠ P, with *h_max_* = 0, *d* = 0. (**a**) Positions; (**b**) Forces.

Finally, referring back to Remark 6, we used the γ-4C controllers defined in Equation (25) with master plant as in Equation (24), except that now the slave model was defined as $P_{s}=\frac{M}{3}s^{2}+2Bs$. The results can be seen in Figure 12. Due to the fact that the models were different, the master and slave forces—obtained from the external forces and local proportional controllers—were also different in order to accomplish position tracking.

Once the performance of the different schemes was analyzed, we studied stability when the time-varying delay was considered. The idea was to maintain as much as possible the ideal tracking properties obtained by the standard controllers in the 4C control scheme by increasing, by means of the γ factor, the stability margin in practical conditions, that is, when there was time-varying delay in signals transmission.

Once the non-delayed part of the master and slave dynamics were fixed (including the gain *K*), only γ remained to be chosen. All the delay uncertainty was characterized by two measures: the maximum delay *h_max_* and the maximum delay rate *d*. 

The stability condition in Equation (20) is based on the $H(s)=:\phi (s)\cdot {\left(I+G(s)\right)}^{-1}G(s)$ system in Equation (21). The closed-loop (non-delayed) system *G*(s) only depended on *γ*, and the “multiplier” *φ* in Equation (19) depended on *h_max_*, *d*. Thus the condition in Equation (20) took the form $\Gamma \left(\gamma ,h_{\max},d\right)<1$. It is important to emphasize that Equation (20) involves the spectral radius of the frequency response *H(jω)* of some *H(s)* depending on (γ, *h_max_*, *d*) in a complex way. Thus, Equation (20) had to be treated numerically (not analytically).

There was an easy way to deal with Equation (20) numerically: the delay uncertainty parameters (*h_max_*, *d*) could be discretized in the ranges of interest, and for each discretized value, the minimum γ could be computed. These computations can be represented in the form of 3D surfaces and the designer can use the numerical results to decide, depending on the expected delay uncertainty, the adequate tuning of γ to be included in the controllers.

**Figure 13:**
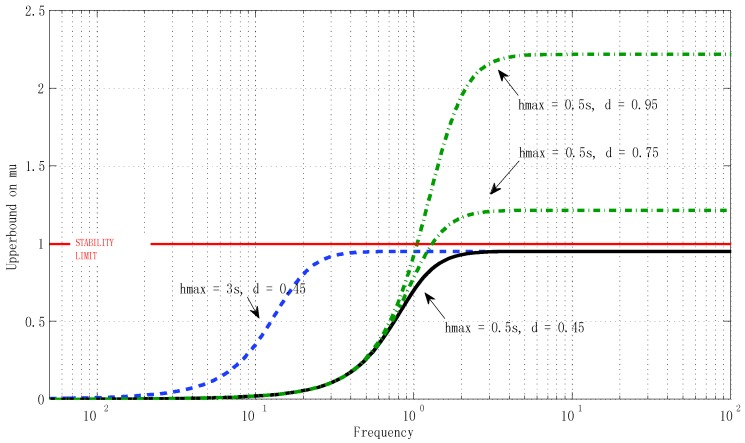
Upper bound on *μ* for *d*, *h*, |*η*(t)| ≤ 0.01.

**Figure 14:**
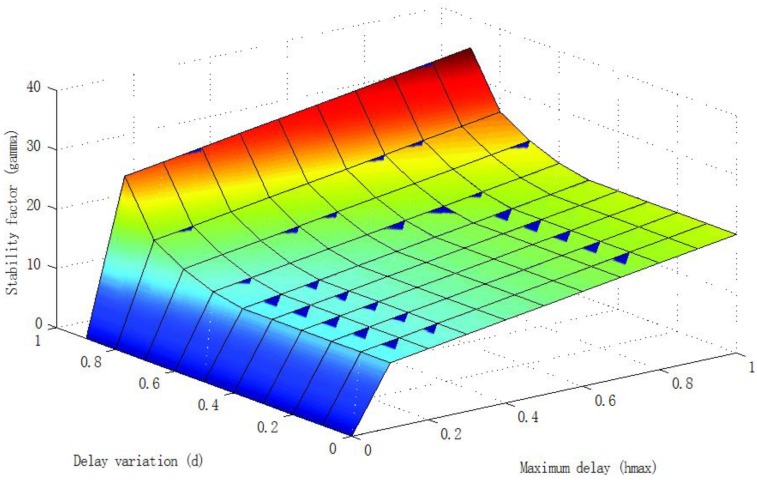
γ_min_ (*z* axis) needed to ensure stability given the delay bounds.

The stability analysis could be assessed applying Theorem 1 and using Matlab. In all cases it had to be proved, firstly, that the system verified the nominal stability condition given in Equation (22). Here we show, briefly as Figure 13, the effect on stability (from the results on SSV) of different bounds on the delay, fixing the stability factor at γ = 12, which was the value selected for the experiments described in the next section. Note that the delay variation was the most critical aspect of stability. The results depicted in Figure 14 show the minimum value of the stability factor γ (*z* axis) needed to ensure the stability of the system given the bounds on the delay (*h_max_*, *d*).

## 6. Experimental Results

In order to test the above simulation results, real experiments were conducted from the haptic teleoperation case study as described above. The Omni devices were connected to the computer through a FireWire connection. The computers contained the local controllers implemented under Matlab/Simulink and Quarc real-time control software as a real-time machine for the experiments. The Quarc software managed the interface with the haptic device using Quarc Phantom Omni software and also collected and saved all exchanged data. The sampling period in the experiments was set to 1 millisecond. The two devices were remotely coordinated under programmed force profiles like the human and environment external forces, and the experimental data validated the stability and performance results in the theoretical part.

**Figure 15:**
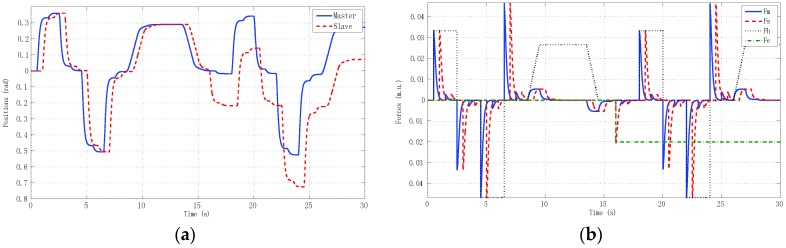
Local teleoperation using linear models of master and slave with *h_max_* = 0.5 s and *d* = 0.45. (**a**) Positions; (**b**) Forces.

**Figure 16:**
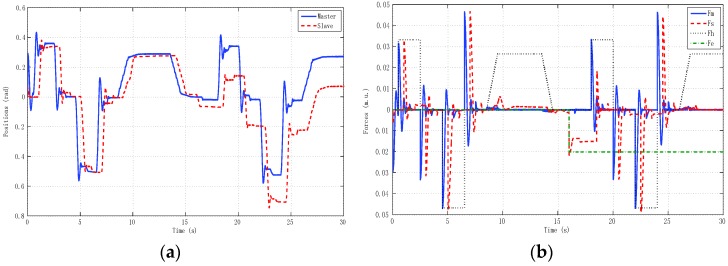
Local teleoperation using haptic devices with *h_max_* = 0.5 s and *d* = 0.45. (**a**) Positions; (**b**) Forces.

We describe results for three experiments. The first experiment (Figure 16) reproduced real local teleoperation of the Omni devices at the University of Vigo, artificially increasing a time-varying delay between master and slave. The simulation case depicted in Figure 15 is compared with this first experiment as depicted in Figure 16.

The results were very similar to the simulation results, although the difference between the linear identified models used in the simulation case and the OMNI nonlinear dynamic with a pre-compensated deadzone Equation (22) can be appreciated. 

**Figure 17:**
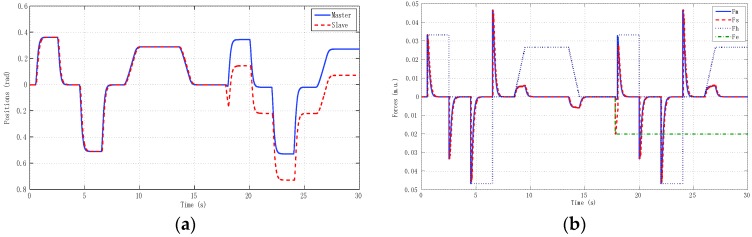
Remote teleoperation between Vigo and Manchester using linear models of master and slave. (**a**) Positions; (**b**) Forces.

**Figure 18:**
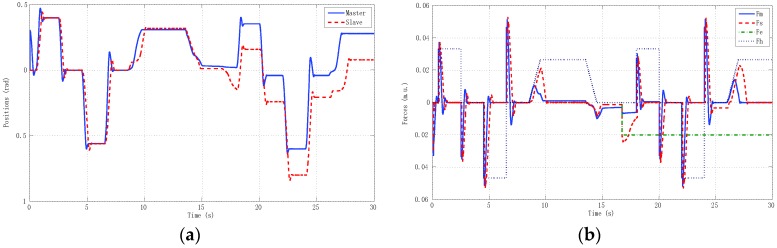
Real remote teleoperation between Vigo and Manchester using haptic devices. (**a**) Positions; (**b**) Forces.

The second experiment (Figure 17) reproduced real remote teleoperation between the University of Vigo and the University of Manchester using linear models of master and slave like the corresponding simulation model described in Section 5. Here we could analyze the impact of time delays separately from model uncertainty and nonlinearities issues. The final experiment (Figure 18) tested real remote teleoperation between the master haptic device located at the University of Vigo and the slave haptic device located at the University of Manchester. Note that, for implementation over the haptic devices, the deadzone was pre-compensated at the input Equation (22) by an inverse function in the form explained in Section 4. In general, this function caused the system to be more oscillating than the linear model and to have a small steady-state error.

**Figure 19:**
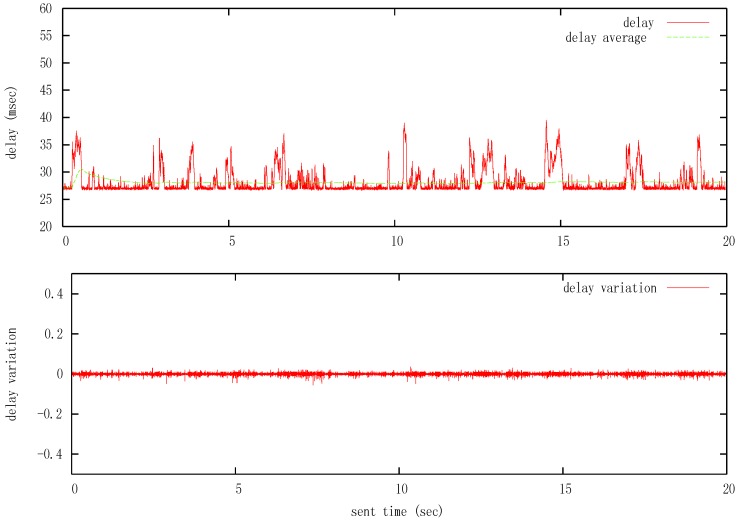
Experimental delay bounds between Vigo and Manchester. (**Top**) Magnitude; (**Bottom**) Variation.

The data (forces and positions) were exchanged between the master and the slave computers by a transmission of UDP packets. Each packet was composed of time stamps and the transmitted value with a fixed size of 68 bytes. Figure 19 shows some experimental data for connections via Internet between Vigo and Manchester. The delay was measured in terms of the round trip time (RTT) communication. The maximum delay was near *h_max_* = 0.028 s, with very low variation tested with a transmission rate of 1000 packets per second (Figure 19a). The test over the communication channel also revealed that variations in time delay never exceeds *d* = 0.45, as can be seen in Figure 19b. It should be noted that the communication network used in the experiment belongs to the GÉANT pan-European research and education network, which has very good quality linkage between facilities.

## 7. Conclusions

In this paper we have extended our generic framework to stability under time-varying delay [11] to scaled 4C or γ-4C teleoperation. The application of the theoretical framework to the four channel case, with time-varying delays, gave rise to an encapsulated delay uncertainty, size 4 × 4 and diagonal. Assuming symmetric delays, the related structured singular value or μ-value was equal to the spectral radius of the linear subsystem. The approach represents an appealing frequency technique to deal with the stability robustness of the architecture. 

The main novelty of the present research compared to previous related research is that we have explicitly discussed the problems associated with ideal transparency and that we have rearrange previous 4C solutions [22,25] so that the resulting controllers are proper, that is, they do not contain single or double differentiators and so avoid problems of noise. We have also validated this research through new simulations and real-life experiments using an Internet teleoperation setup that coordinates two Omni haptic devices between remote laboratory setups in Spain (University of Vigo) and the UK (University of Manchester).

The experiments achieved remote device coordination under programmed force profiles. This required resolving a large number of practical issues related to hardware, software and communications. Some important practical problems were related to linearized model identification and to deadzone or static friction in the Omni devices, which had to be pre-compensated. Finally, the architecture was fine-tuned and successfully tested, obtaining complete stability to zero of the whole state and robustness against continuous-time time-varying delay for Internet-based teleoperation. The real and simulated experiments give similar results, thus validating the models. Stable remote teleoperation also performed adequately in terms of position synchronization and force transparency.

This research has several possible extensions to future work. First, time-varying delay uncertainty was treated here by means of encapsulation [29], but exploring other novel approaches to uncertain time delays could improve the robustness analysis, for example, Lyapunov-Krasovskii functionals [32]. The generic framework based on IQCs [33] is also a potentially powerful tool in addressing teleoperation robustness. Second, one of the main difficulties encountered was the problem of deadzones as, although the coarse values of the (asymmetrical) deadzone widths can easily be estimated, the related static friction does not repeat well from one experiment to another. Since deadzone is a static monotone bounded uncertainty, it could potentially be addressed by means of the IQC approach that uses Zames-Falb multipliers to deal with robust stability [34].

## Figures and Tables

**Table 1:** Control Schemes (P_m_ = P_s_ = P = 0.001s^2^ + 0.02s, C_m_ = C_s_ = K = 0.1, C_5_ = C_6_ = K’ = 0).

	C_1_	C_2_	C_3_	C_4_	$\Delta_{\Lambda }$	$\Delta_{\Lambda }\Lambda$
PE	K	0	0	−K	$P^{2}+2PK$ s_i_ = 0, −20, −10 ± 10i	$\left(\begin{array}{cc} P+K & K\\ K & P+K \end{array}\right)\;$
FP	0	0	1	−K	$P^{2}+PK+K^{2}$ s_i_ = −18.7 ± 5i, −1.3 ± 5i	$\left(\begin{array}{cc} P+K & K\\ P & P+K \end{array}\right)\;$
PF	K	−1	0	0	$P^{2}+PK+K^{2}$ s_i_ = −18.7 ± 5i, −1.3 ± 5i	$\left(\begin{array}{cc} P+K & P\\ K & P+K \end{array}\right)\;$
3C-i	K	−1	0	−K	$P^{2}+PK$ s_i_ = 0, −20, −10, −10	$\left(\begin{array}{cc} P+K & P+K\\ K & P+K \end{array}\right)\;$
3C-ii	K	0	1	−K	$P^{2}+PK$ s_i_ = 0, −20, −10, −10	$\left(\begin{array}{cc} P+K & K\\ P+K & P+K \end{array}\right)\;$
4C (γ = 1.07)	K	−1/γ	1	−K/γ	${\left(P+K\right)}^{2}\cdot \left(1-\frac{1}{\gamma }\right)$ s_i_ = −10, −10, −10, −10	$\left(\begin{array}{cc} P+K & \frac{1}{\gamma }(P+K)\\ P+K & P+K \end{array}\right)\;$

## Appendix A

In the proposed scheme the master and slave are modeled as mechanical systems given by the following equation in Laplace domain Equation (A1):

(A1) $$P_{m}(s)X_{m}(s):=F_{m}(s),\;P_{s}(s)X_{s}(s):=F_{s}(s)$$

The output vector of the plant *y_m_* = (x_m_ f_m_)^T^ that sends information to the slave in the 4C scheme is a *n_y_* × 1 = 2 × 1 vector because the system exchanges both positions and forces. It can be expressed in Laplace domain as a function of the scalar *X_m_*:

(A2) $$Y_{m}(s)=\left(D_{1m}+D_{2m}s+D_{3m}P_{m}(s)\right)X_{m}(s)=:D_{m}(s)X_{m}(s)\;\Rightarrow D_{m}=\left(\begin{array}{c} 1\\ P_{m} \end{array}\right),\;D_{1m}=\left(\begin{array}{c} 1\\ 0 \end{array}\right),\;D_{2m}=\left(\begin{array}{c} 0\\ 0 \end{array}\right),\;D_{3m}=\left(\begin{array}{c} 0\\ 1 \end{array}\right)$$

The other output of the plant *z_m_*, used by the local controller, is a *n_z_* = 1 signal which is the position. Hence:

(A3) $$Z_{m}(s)=\left(E_{1m}+E_{2m}s\right)X_{m}(s)=:E_{m}(s)X_{m}(s)\;\Rightarrow E_{m}=\left(\begin{array}{cc} 1 & 0 \end{array}\right)$$

A generic controller in the Laplace domain that uses the previous notation (see Figure 1) can be described by:

(A4) $$F_{mc}(s)=C_{1m}(s)Z_{m}(s)+C_{2m}(s)Y_{sd}(s)+C_{3m}(s)F_{h}(s)$$

where the values of *C_im_* can be obtained by comparing the master controller in 4C as expressed in (5) with Equation (A4).

(A5) $$C_{1m}=C_{m},\;C_{2m}=\left(\begin{array}{cc} C_{4} & C_{2} \end{array}\right),\;C_{3m}=-C_{6}$$

Combining all the previous equations, and omitting, for simplicity sake, dependence on *s,* we obtain:

(A6) $$P_{m}X_{m}=F_{m}=F_{h}-F_{mc}=F_{h}-C_{1m}E_{m}X_{m}-C_{2m}Y_{sd}-C_{3m}F_{h},\;\left(P_{m}+C_{1m}E_{m}\right)X_{m}=-C_{2m}Y_{sd}+\left(I-C_{3m}\right)F_{h}$$

On the slave side, the environment force is *f_e_*, whereas the controller force is *f_sc_* and thus $f_{s}=f_{e}+f_{sc}.$ Hence:

(A7) $$\left(P_{s}-C_{1s}E_{s}\right)X_{s}=C_{2s}Y_{md}+\left(I+C_{3s}\right)F_{e}\;C_{1s}=-C_{s},\;C_{2s}=\left(\begin{array}{cc} C_{1} & C_{3} \end{array}\right),\;C_{3s}=C_{5},\;E_{s}=\left(\begin{array}{cc} 1 & 0 \end{array}\right)\;D_{s}=\left(\begin{array}{c} 1\\ P_{s} \end{array}\right),\;D_{1s}=\left(\begin{array}{c} 1\\ 0 \end{array}\right),\;D_{2s}=\left(\begin{array}{c} 0\\ 0 \end{array}\right),\;D_{3s}=\left(\begin{array}{c} 0\\ 1 \end{array}\right)$$

In compact form, with 0_r × c_ as a zero matrix with *r* rows and *c* columns *s*:

(A8) $$\left(\begin{array}{cc} P_{m}+C_{1m}E_{m} & 0\\ 0_{} & P_{s}-C_{1s}E_{s} \end{array}\right)\cdot \left(\begin{array}{c} X_{m}\\ X_{s} \end{array}\right)=-\left(\begin{array}{cc} 0_{1\times n_{y}} & C_{2m}\\ -C_{2s} & 0_{1\times n_{y}} \end{array}\right)\cdot \left(\begin{array}{c} Y_{md}\\ Y_{sd} \end{array}\right)\;+\left(\begin{array}{cc} I-C_{3m} & 0\\ 0 & I+C_{3s} \end{array}\right)\cdot \left(\begin{array}{c} F_{h}\\ F_{e} \end{array}\right)$$

That is:

(A9) $$\Pi (s)X(s)=-C(s)Y_{d}(s)+\Phi (s)F(s)$$

Now *Y_d_* = (*Y_md_ Y_sd_*)^T^ can be obtained as a function of *Y* = (*Y_m_ Y_s_*)^T^ by applying the time-delay expressions in Equations (2)–(4), with *I_ny_* as the *n_y_* = 2 order identity matrix:

(A10) $$\left(\begin{array}{c} Y_{md}\\ Y_{sd} \end{array}\right)=\left(\begin{array}{cc} I_{n_{y}}\cdot D_{m} & 0_{n_{y}\times n_{y}}\\ 0_{n_{y}\times n_{y}} & I_{n_{y}}\cdot D_{s} \end{array}\right)\cdot \left(\begin{array}{c} Y_{m}\\ Y_{s} \end{array}\right),\;\text{or}\;\;Y_{d}=DY,$$

Considering Equation (A2) and the corresponding slave equation, we obtain:

(A11) $$\left(\begin{array}{c} Y_{m}\\ Y_{s} \end{array}\right)=\left(\begin{array}{cc} D_{m} & 0_{n_{y}\times 1}\\ 0_{n_{y}\times 1} & D_{s} \end{array}\right)\cdot \left(\begin{array}{c} X_{m}\\ X_{s} \end{array}\right),\;\;\text{or}\;Y=DX$$

Finally, replacing Equations (A10) and (A11) in Equation (A9), we have a complete description of the system:

(A12) Π(s)X(s)=−C(s)DD(s)X(s)+Φ(s)F(s)

## Appendix B

Here we reproduce the classical implementation of the 4C control scheme, but maintaining the sign conventions as in Figure 1, that is, with the same definition of $f_{m}=f_{h}-f_{mc},\;f_{s}=f_{e}+f_{sc}.$ In this case, the master and slave systems exchange external forces *f_e_* and *f_h_*, instead of *f_m_* and *f_s_* in our proposal. Accordingly, Equation (5) results as follows:

(B1) $$f_{mc}=C_{m}x_{m}+C_{2}f_{ed}+C_{4}x_{sd}-C_{6}f_{h}\;f_{sc}=-C_{s}x_{s}+C_{3}f_{hd}+C_{1}x_{md}+C_{5}f_{e}$$

and the addmitance matrix definition for zero delay is now given by:

(B2) $$\Lambda ={\left(\Pi +CD\right)}^{-1}\Phi ={\left(\begin{array}{cc} P_{m}+C_{m} & C_{4}\\ -C_{1} & P_{s}+C_{s} \end{array}\right)}^{-1}\cdot \left(\begin{array}{cc} 1+C_{6} & -C_{2}\\ C_{3} & 1+C_{5} \end{array}\right)$$

(B3) $$\left(\begin{array}{c} x_{m}\\ x_{s} \end{array}\right)=\Lambda \cdot \left(\begin{array}{c} f_{h}\\ f_{e} \end{array}\right)\Rightarrow \left\{ \begin{array}{l} x_{m}=\Delta_{\Lambda }{}^{-1}\cdot (\Lambda_{11}f_{h}+\Lambda_{12}f_{e})\\ x_{s}=\Delta_{\Lambda }{}^{-1}\cdot (\Lambda_{21}f_{h}+\Lambda_{22}f_{e}) \end{array}\right.$$

with the following values:

(B4) $$\Lambda_{11}=\left(P_{s}+C_{s}\right)\cdot \left(1+C_{6}\right)-C_{4}C_{3}\;\Lambda_{12}=-\left(P_{s}+C_{s}\right)C_{2}-C_{4}\left(1+C_{5}\right)\;\Lambda_{21}=C_{1}\left(1+C_{6}\right)+C_{3}\left(P_{m}+C_{m}\right)\;\Lambda_{22}=-C_{1}C_{2}+\left(P_{m}+C_{m}\right)\cdot \left(1+C_{5}\right)$$

(B5) $$\Delta_{\Lambda }=\left(P_{m}+C_{m}\right)\cdot \left(P_{s}+C_{s}\right)+C_{1}C_{4}$$

The transparency conditions are achieved by selecting:

(B6) $$For\;C_{m},C_{s}:C_{4}(s)=-\left(P_{m}+C_{m}\right),\begin{array}{cc} & C_{1}(s)=P_{s}+C_{s}, \end{array}\;\begin{array}{cccc} C_{2}=-\left(1+C_{6}\right), & C_{3}=1+C_{5} & & \end{array}$$

Note that for $\Delta_{\Lambda }\to 0$, C_1_ and C_4_ must be not proper.
